# Prenatal sonographic characteristics and postnatal outcomes of congenital portosystemic shunt diagnosed during the fetal period: a systematic review

**DOI:** 10.1186/s13023-025-03811-3

**Published:** 2025-05-27

**Authors:** Bo Kong, Xiaoyi Yan, Yang Gui, Tianjiao Chen, Hua Meng, Ke Lv

**Affiliations:** Department of Ultrasound, Peking Union Medical College, Peking Union Medical College Hospital, Chinese Academy of Medical Sciences, No. 1, Shuai Fu Yuan, Dong Cheng District, Beijing, 100730 China

**Keywords:** Abernethy malformation, Prenatal, Ultrasound, Umbilical-portal-systemic venous shunt

## Abstract

**Background:**

Congenital portosystemic shunt (CPSS) is a rare vascular malformation which results in anomalous communication between the portal venous system and the systemic vein. The objective of this review is to describe the prenatal ultrasonic characteristics and postnatal outcomes of CPSS diagnosed prenatally, along with providing some suggestions for perinatal monitoring.

**Materials and methods:**

A systematic literature search was conducted in PubMed and Ovid electronic databases in a period from January 2012 to May 2024, using the terms: “congenital portosystemic shunt”, “ductus venosus”, “Abernethy malformation” and “umbilical-portal-systemic venous shunt”. All original studies reporting CPSS patients diagnosed prenatally were included. Studies published in languages other than English or studies that did not report the clinical information of patients were excluded. Two reviewers independently screened articles for inclusion and extracted data.

**Results:**

A total of 39 studies which included 525 cases were enrolled in the systematic review. Among the included patients, 49 (9%) had umbilical-systemic shunt (USS), 264 (50%) had ductus venosus-systemic shunt (DVSS), 159 (30%) had intrahepatic portal-systemic shunt (IHPSS), 32 (6%) had extrahepatic portal-systemic shunt (EHPSS) and 9 (2%) had mixed shunts which meant that they had more than one type of shunts. There were also 12(3%) patients were excluded because the specific type was not described in the study. CPSS patients could have severe comorbidities such as chromosomal aberrations and cardiovascular malformations. Compared with other groups, fetuses with IHPSS had the lowest incidence of severe comorbidities. Most patients received conservative treatment while interventional and surgical treatments were used for some patients.

**Conclusion:**

We suggest that fetuses diagnosed with CPSS should be treated differently according to their types and clinical manifestations. IHPSS patients are more likely to have good outcomes so they may benefit from the “wait-and-see” approach while the other groups deserve closer monitoring. Personalized treatment is essential as CPSS patients can be asymptomatic or have severe complications.

## Background

Congenital portosystemic shunt (CPSS), first reported in 1793 [1], is a rare vascular malformation. The incidence of CPSS is approximately one case per 30,000–50,000 children [2]. Owing to the false evolution or involution of vitelline veins during embryogenesis, CPSS results in anomalous communication between the portal venous system and the systemic vein, which often occurs with other comorbidities such as cardiomegaly, intrauterine growth restriction (IUGR), chromosomal aberrations and encephalopathy [3–7]. Several classifications of CPSS have been established [8–10]. For example, one type of classification was proposed by Stringer [11] in 2008 in which CPSS was divided into two subtypes: extrahepatic porto–systemic shunt (EHPSS) and intrahepatic porto–systemic shunt (IHPSS). These classifications can aid clinicians in obtaining a better understanding and more precisely predicting outcomes of CPSS. However, these classifications cannot fully meet clinical needs, and most of the existing information regarding the diagnosis, clinical symptoms and significance of CPSS derives from pediatric and adult studies [12].

Unlike in adults, there exists three venous systems in the fetuses, including the umbilical vein, portal vein and ductus venosus, which form a functionally inseparable blood vessel transporting the highly oxygenated blood from the placenta to the left atrium [2, 12]. Abnormal development of any of the three venous systems can lead to shunting into the systemic veins, termed umbilical–portal–systemic venous shunt (UPSVS). With the closure of the umbilical vein and ductus venosus after birth, the postnatal classifications of shunts lack two components of the essential venous complex, and refer only to survivors; therefore, these CPSS classification systems are not applicable for fetuses.

A new in-utero classification based on the anatomical origin of the shunt was proposed in 2016 [12]. Taking into account the in-utero umbilical vein-portal vein-ductus venosus (UV-PV-DV) structure as an intact system, the UPSVS was classified into 3 types: Type I, umbilical–systemic shunt (USS); Type II, ductus venosus–systemic shunt (DVSS); and Type III, portal–systemic shunt, which was divided into two subgroups: Type IIIa, IHPSS; and Type IIIb, EHPSS. Each type had its own special postnatal clinical characteristics. For example, IHPSS patients might experience spontaneous closure in the first few months of life and have no symptoms.

Although the number of CPSS patients diagnosed prenatally is increasing, studies on the prenatal diagnosis of CPSS are scarce, warranting further studies to expand our knowledge to improve perinatal management and counselling since early diagnosis and appropriate intervention are important to avoid deterioration of the disease [13, 14]. Here, we presented a systematic review of the studies on CPSS, including only patients who were diagnosed prenatally, with the aim of describing their prenatal ultrasonic characteristics and postnatal outcomes, along with proposing some appropriate strategies for the perinatal management and counselling regarding CPSS.

## Methods and materials

### Data search and eligibility criteria

The review was planned and carried out according to the recommendations of the Preferred Reporting Items for Systematic Review and Meta-analysis (PRISMA) Statement [15]. Based on previously conducted studies, our study was a review of the associations between prenatal sonographic characteristics and postnatal outcomes of CPSS. An electronic search was performed in PubMed and Ovid databases for studies reporting CPSS patients diagnosed prenatally from January 2012 to May 2024, using the terms: “congenital portosystemic shunt”, “ductus venosus”, “Abernethy malformation” and “umbilical-portal-systemic venous shunt”. The search was conducted separately for each database and the keyword search was limited to titles and abstracts. The last search was conducted on June 10, 2024.

### Inclusion and exclusion criteria

All published literatures related to the prevalence, diagnosis, management or outcomes of CPSS were included. The exclusion criteria were as follows:

(1) studies that comprised only reviews, systematic reviews, editorial letters or conference abstracts; (2) studies published in languages other than English; (3) experimental animal studies; (4) studies that reported only cases of CPSS diagnosed postnatally; and (5) studies that did not report the clinical information of the patients.

The criteria were applied in two steps: first, studies were screened by titles and abstracts for relevance. Second, the full texts of studies, that seemed potentially relevant, were assessed for inclusion.

### Study selection and data extraction

All studies identified during the electronic search were screened and extracted by two independent reviewers. For each included study, we recorded the following data: type of CPSS according to the new classification put forward by Achiron and Kivilevitch [12], maternal age, gestational age at diagnosis, number of patients, prenatal imaging findings, fetal outcomes (termination of pregnancy, death or survival), postnatal clinical symptoms, associated comorbidities and treatment (where available). All the information needed was determined before the review was started. No assumptions were made during the process of data collection and all the collected information was clearly stated in the original reports. The information extracted from the included reports was entered into predesigned sheets (using Excel 2019).

### Quality assessment

Included studies were reviewed and evaluated independently by two authors. To improve the reliability of this analysis, both authors would discuss when discrepancy was found, and consensus agreement was reached.

### Statistical analysis

Results were presented by absolute frequencies and percentages for quantitative variables. For assessment of differences between all groups and between particular groups, we used analysis of the chi-square test or Fisher’s precision probability test and post-hoc Bonferroni tests (using SPSS 24.0). All tests were two-tailed and a *P*-value < 0.05 was considered statistically significant.

## Results

**Fig. 1:**
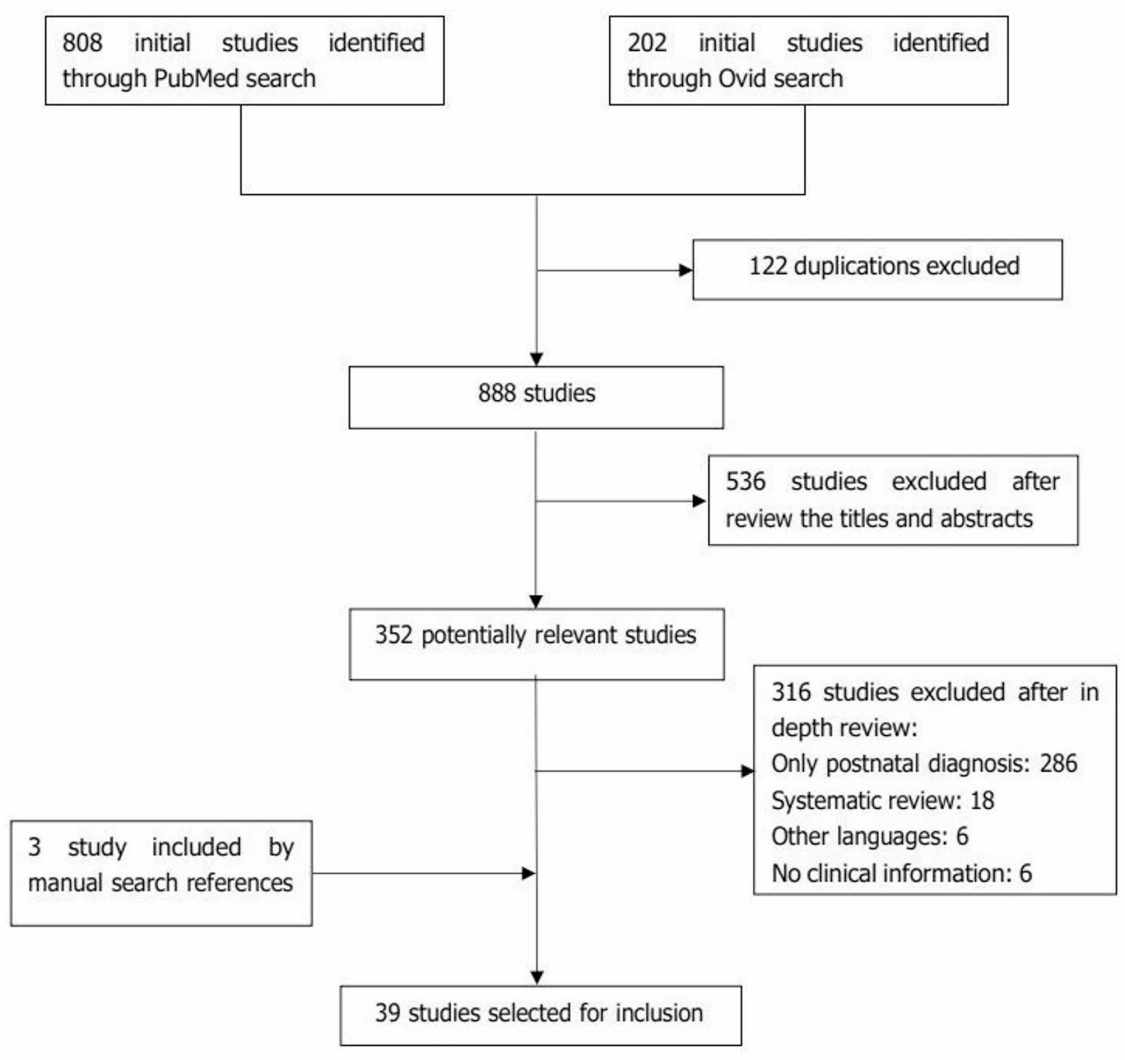
The diagram of the publication retrieval and screening process

A total of 808 and 202 records were retrieved from the PubMed and Ovid databases, respectively. All the studies identified during the electronic search were alphabetically ordered and 122 duplicates were excluded. The remaining 888 studies were subsequently screened by two independent authors. A total of 536 papers were deemed irrelevant after title and abstract screening, and 352 underwent full text review. A total of 39 studies were ultimately included. A diagram of the publication retrieval and screening process was shown in Fig. 1.

The number of cases reported each year was shown in Table 1. A total of 525 CPSS patients were screened for data extraction. All the patients included were diagnosed with CPSS prenatally by ultrasound examinations. Among the included patients, 49 (9%) had umbilical-systemic shunt (USS), 264 (50%) had ductus venosus-systemic shunt (DVSS), 159 (30%) had intrahepatic portal-systemic shunt (IHPSS), 32 (6%) had extrahepatic portal-systemic shunt (EHPSS) and 9 (2%) had mixed shunts which meant that they had more than one type of shunts. Twelve (2%) patients were excluded because the specific type was not described in the original study.

**Table 1:** The number of cases reported of each year

Year	2012	2013	2014	2015	2016	2017	2018	2019	2020	2021	2022	2023	2024	Total
Studies	3	3	3	3	3	1	1	3	6	5	5	2	1	39
Cases	29	6	22	13	60	12	1	130	40	103	68	27	14	525

### Outcome

The outcomes of the 525 patients were summarized in Table 2. The gestational age at diagnosis ranged from 11 weeks to 40 weeks and the maternal age ranged from 20 to 48 years old. In our study, “death” (including intrauterine death and death during the follow-up period) was considered as a poor outcome and “survival” (meaning that the patient was alive during the follow-up period) was considered as a good outcome. Our review revealed that 53 patients had poor outcomes and 324 patients had good outcomes. Three IHPSS patients and two USS patients were lost to follow up. The IHPSS group had the highest rate of good outcomes (134/159, 84%), followed by the DVSS (159/264, 60%), EHPSS (18/32, 56%), mixed shunts (4/9, 45%) and USS groups (9/49, 18%).

**Table 2:** The outcomes of collected cases

Type	Cases	GA (weeks)	Survival	TOP	Death
USS	49	11–38	9 (18%)	31 (63%)	7 (14%)
DVSS	264	11–37	159 (60%)	67 (25%)	38* (14%)
IHPSS	159	15–40	134 (84%)	17 (11%)	5 (3%)
EHPSS	32	21–38	18 (56%)	14 (44%)	0 (0)
Mixed shunts	9	28–38	4 (45%)	2 (22%)	3 (33%)

3 IHPSS cases and 2 USS cases were lost to follow up

GA: gestational age at diagnosis; TOP: termination of pregnancy; *: one case had a spontaneous abortion

USS: Umbilical–systemic shunt; DVSS: Ductus venosus–systemic shunt; IHPSS: Intrahepatic porto–systemic shunt; EHPSS: Extrahepatic porto–systemic shunt

### Main comorbidities

The most frequent comorbidities of CPSS and their cases were summarized in Table 3, including intrauterine growth restriction (IUGR, 100/369, 27%), chromosomal aberrations (87/382, 23%), cardiomegaly (49/483, 10%), cardiovascular malformations (117/483, 24%) and other malformations (89/483, 18%). The most common chromosomal aberration was Trisomy 21 (43/87, 49%), followed by Turner Syndrome (10/87, 11%) and Trisomy 18 (6/87, 7%). Ventricular septal defect (VSD) was the most common cardiovascular malformation (32/117, 27%), followed by atrial septal defect (ASD, 17/117, 15%), left ventricular hypoplasia (15/117, 13%), aortic coarctation (7/117, 6%), patent ductus arteriosus (PDA, 5/117, 4%) and hypertrophic cardiomyopathy (HCM, 4/117, 3%). Compared with the other groups, the IHPSS group presented the highest incidence of IUGR (73/126, 58%), EHPSS group presented the highest incidence of chromosomal aberrations (5/9, 56%), USS group presented the highest incidence of cardiovascular malformations (19/49, 39%) and other malformations (20/49, 41%). The group with mixed shunts presented the highest incidence of cardiomegaly (4/9, 44%).

**Table 3:** Comparisons of the incidence of main comorbidities between different types

Type	USS (Type I)	DVSS (Type II)	IHPSS (Type IIIa)	EHPSS (Type IIIb)	Mixed shunts	P*
Sum	49	264	159	32	9	
IUGR	5 (15%,*n* = 34)	18 (10%,*n* = 187)	73 (58%,*n* = 126) IHPSS vs. DVSS *P* < 0.001	3 (21%,*n* = 14)	1 (13%,*n* = 8)	< 0.001
Chromosomal Aberrations (Trisomy21 + other)	9 + 4 (33%,*n* = 39) USS vs. IHPSS *P* = 0.003	30 + 25 (24%,*n* = 228) DVSS vs. IHPSS *P* = 0.020	3 + 10 (13%,*n* = 101)	0 + 5 (56%,*n* = 9) EHPSS vs. IHPSS *P* = 0.006	1 + 0 (20%,*n* = 5)	0.007
Cardiomegaly	6 (12%,*n* = 49)	27 (11%,*n* = 254)	9 (6%,*n* = 149)	3 (14%,*n* = 22)	4 (44%,*n* = 9) Mixed vs. IHPSS *P* = 0.003	0.030
Cardiovascular Malformations	19 (39%,*n* = 49) USS vs. IHPSS *P* < 0.001	75 (30%,*n* = 254) DVSS vs. IHPSS *P* < 0.001	13 (9%,*n* = 149)	8 (36%,*n* = 22) EHPSS vs. IHPSS *P* = 0.001	2 (22%,*n* = 9)	< 0.001
Other Malformations	20 (41%,*n* = 49) USS vs. IHPSS *P* < 0.001	47 (19%,*n* = 254)	17 (11%,*n* = 149)	2 (9%,*n* = 22)	3 (33%,*n* = 9)	< 0.001

P*: the result of the chi-square test or Fisher’s precision probability test among all groups; Other malformations: urinary malformation, neurologic malformation, orthopedic malformation and so on; Other Chromosomal Aberrations: trisomy 18, turner syndrome and so on; USS: umbilical–systemic shunt; DVSS: ductus venosus–systemic shunt; IHPSS: intrahepatic porto–systemic shunt; EHPSS: extrahepatic porto–systemic shunt; IUGR: intrauterine growth restriction;

A comparison of the incidence of chromosomal aberrations in CPSS patients with and without cardiovascular malformations was also conducted, since cardiovascular malformations might be caused by chromosomal aberrations. The statistics were shown in the Table 4. A total of 178 cases were included because their karyotype test and echocardiography results were simultaneously described, of whom 14 patients had both chromosomal aberrations and cardiovascular malformations, 27 patients had only chromosomal aberrations, 31 patients had only cardiovascular malformations and 106 patients had neither condition. The results of the chi-square test were not statistically significant.

**Table 4:** The comparison of the incidence of chromosomal aberration in CPSS patients with and without cardiovascular malformations

	CPSS patients with Chromosomal aberrations		CPSS patients without Chromosomal aberrations	*P*
CPSS patients with cardiovascular malformations	14	31		0.137
CPSS patients without cardiovascular malformations	27	106		

CPSS: congenital portosystemic shunt

### Postnatal clinical symptoms and treatment

The postnatal clinical symptoms and treatment reported in the original studies were summarized in Tables 5 and 6. Postnatal clinical symptoms were reported for 141 patients. Among these patients, 84 neonates were asymptomatic and 57 neonates had clinical symptoms, including jaundice (23/141, 16%), hyperammonemia (23/141, 16%), elevated transaminase levels (15/141, 11%), hepatosplenomegaly (12/141, 9%), hypoglycemia (6/141, 4%) and cholestasis (2/141, 1%). Asymptomatic patients referred to those exhibited no clinical manifestations associated with CPSS during the follow-up period after birth. Regarding treatment, 246 neonates received conservative treatment, 31 neonates received interventional or surgical treatment and the treatment types for the remaining patients were not reported in the original studies. According to the results of the statistical analysis, the neonates diagnosed with EHPSS had the highest incidence of postnatal clinical symptoms and they were more likely to need interventional or surgical treatment to close the shunts.

**Table 5:** Comparisons of the postnatal clinical manifestations between different types

Type	USS (Type I)	DVSS (Type II)	IHPSS (Type IIIa)	EHPSS (Type IIIb)	Mixed Shunts	*P*
Asymptomatic neonates	5	20	57	1	1	0.002
Symptomatic neonates	2	6 DVSS vs. EHPSS *P* = 0.001	37 IHPSS vs. EHPSS *P* = 0.005	9	3	

USS: Umbilical–systemic shunt; DVSS: Ductus venosus–systemic shunt; IHPSS: Intrahepatic porto–systemic shunt; EHPSS: Extrahepatic porto–systemic shunt

**Table 6:** Comparisons of the choice of treatment between different types

Type	USS (Type I)	DVSS (Type II)	IHPSS (Type IIIa)	EHPSS (Type IIIb)	Mixed Shunts	*P*
Conservative treatment	2	122	113	8	1	< 0.001
Interventional or Surgical treatment	1	5 DVSS vs. EHPSS *P* < 0.001	13 IHPSS vs. EHPSS *P* < 0.001	10	2	

USS: Umbilical–systemic shunt; DVSS: Ductus venosus–systemic shunt; IHPSS: Intrahepatic porto–systemic shunt; EHPSS: Extrahepatic porto–systemic shunt

## Discussion

CPSS, which is seldom observed in the clinical practice, is a malformation leading to anomalous communication between the portal venous system and the systemic vein. Compared with previous classifications, the in-utero classification is more comprehensive and appropriate for describing fetal CPSS as it is based on the fetal blood circulation pathway which is different from that in adults. Ultrasound examination allows noninvasive monitoring throughout the prenatal and postnatal periods, which can protect children from radiation damage and early over-intervention. Abnormal vascular connections detected by ultrasonography are direct signs of CPSS. In addition, other ultrasonic signs such as an anomalous tubular structure, a prominent hepatic vein and a prominent umbilical vein can be useful for diagnosing CPSS [2]. Some patients may have prenatal comorbidities, such as IUGR, and these comorbidities will attract attention of obstetricians, which can be helpful for the detection of CPSS. Early prenatal diagnosis may significantly influence the clinical outcomes because it is helpful to monitor the progression of the disease so that essential treatment can be provided in time [4]. We performed a search of studies including CPSS patients diagnosed antenatally to provide some suggestions for perinatal monitoring of CPSS.

On the basis of our review, the incidence of each comorbidity varied by type, so there was a difference in the rate of good outcomes across the types. IHPSS patients had a lower incidence of severe comorbidities such as cardiovascular malformations and chromosomal aberrations, which could explain why fetuses with IHPSS were more likely to have better outcomes. In contrast, the other groups had a higher incidence of severe comorbidities, especially the USS group, which had the lowest rate of good outcomes. Previous studies demonstrated that hydrops more often co-occurred with associated cardiac and chromosomal abnormalities, which might result in intrauterine death [16–22]. Consequently, the fetuses with USS deserved close monitoring after diagnosis.

IHPSS is defined as a shunt between the intrahepatic portal venous system and hepatic veins, and IHPSS patients tend to have a normal intrahepatic portal venous system (IHPVS) so they have better hemodynamic tolerance and better outcomes [13]. Achiron et al. [12] demonstrated that the integrity of the IHPVS played a pivotal role as a prenatal prognostic factor and in optimizing postnatal care. Consequently, although our review revealed that IHPSS group had the highest incidence of IUGR, it also had the highest rate of good outcomes.

IUGR is commonly observed in CPSS patients, and is a marker of poor fetal hemodynamics [23–25]. According to previous studies, the liver blood perfusion decreased in CPSS patients, which resulted in the reduction in insulin-like growth factor-I and II mRNA expression that led to decreased peripheral tissue growth [2, 19, 26–29]. Therefore, the volume of the shunts might be another prognostic factor. Our review also revealed that patients with mixed shunts had the highest rate of poor outcomes. IUGR can also be caused by other factors such as chromosomal aberrations, fetal infection, and placental insufficiency. Therefore, in patients with IUGR of unknown etiology, the potential diagnosis of CPSS should be considered.

The prevalence of cardiac malformations in CPSS patients is dramatically higher than that in the general population [30]. Because the shunts can increase heart preload and potentially cause heart overload [31, 32]. Unlike IHPSS, the USS, DVSS and EHPSS, which are from umbilical vein, ductus venosus and portal vein, respectively, are characterized by drainage into systematic veins such as inferior vena cava (IVC). The shunts can also drain into the right atrium directly, causing the heart preload to be significantly greater and the hemodynamic change to be worse [13, 18, 22, 33, 34]. Our review showed that the incidence of cardiovascular malformations in these groups was higher than that in the IHPSS group. Cardiovascular malformations might be also caused by chromosomal aberrations, but according to our data, the occurrence of cardiovascular malformations in CPSS patients was not associated with chromosomal aberrations. There was also a hypothesis describing the links between cardiovascular malformations and CPSS that hemodynamic changes secondary to cardiac defects might affect the persistence of vitelline veins during embryonic development of the portal system, and contributed to the development or maintenance of CPSS [30]. Thus, fetal echocardiography should be conducted when CPSS is diagnosed. Close monitoring is important if the fetuses have cardiovascular malformations. More attention should be given if the type of the shunt is USS, DVSS or EHPSS.

Chromosomal aberration is another comorbidity that often occurs in CPSS patients [35, 36]. Specifically, although chromosomal aberrations could occur in fetuses with all types, they were mostly associated with USS, DVSS and EHPSS fetuses in our review. Most fetuses with chromosomal aberrations had disappointing outcomes. Their parents were more likely to terminate the pregnancies, which was one of the reasons for the lower rates of good outcomes in USS, DVSS and EHPSS groups. Although the link between chromosomal aberrations and CPSS has not been fully understood, this discovery is also meaningful for perinatal counselling and antenatal examination. Since the fetuses diagnosed with USS, DVSS and EHPSS tend to have chromosomal aberrations, chromosome examination or genetic examination is necessary for these patients.

Postnatal clinical symptoms are crucial factors in the choice of treatment. In our review, more than half of the patients were asymptomatic, and most of them were diagnosed with DVSS and IHPSS. It was reported that the shunts could close spontaneously before two years old particularly in children with IHPSS [4, 37–42]. There were several pathophysiological mechanisms used to explain this phenomenon: the pressure in the right atrium would decrease due to the permeabilization of the pulmonary circulation or the closure of umbilical vein after birth, which might reduce the pressure in the liver area and force blood to flow out of the shunts to other portal branches so that the venous system of the shunts collapsed and eventually closed [29, 37]. Some DVSS patients had similar situations, the ductus venosus closed and gradually degenerated due to decreased blood flow and circulating prostaglandins after birth [43–45]. Therefore, conservative treatment tends to be the first choice.

In our review, only 31 neonates received interventional or surgical treatment to close their shunts. However, each patient should receive personalized treatment, since some patients could have severe clinical manifestations and should be treated. The diversion of metabolites and vasoactive mediators, such as ammonia and bile acid, from the splanchnic venous system directly into the systemic circulation, along with the reduced liver blood perfusion, had an adverse effect on metabolism. These substances accumulated within the body and led to the corresponding symptoms. In neonates, these metabolites and the vasoactive mediators could also cause damage to their growing brains [6, 46–48]. Therefore, the volume of the shunt, which could be influenced by CPSS type, was another factor affecting the clinical manifestations and outcomes. It has been proposed that if the shunt does not close spontaneously and remains patent in the second year of life, or if severe clinical symptoms are present (such as hyperglycemia, jaundice and liver injury) or there is a large shunt flow, surgical or endovascular intervention is required [6, 49]. Neonates with EHPSS tend to have postnatal clinical symptoms so their physical conditions should be monitored so that essential treatment can be provided in a timely manner.

There were still limitations to our study. First, our study was a retrospective review and it was difficult for us to obtain the original clinical statistics and sonographies. The clinical information we were interested in might not have been fully described in the original studies. Second, since there were different ways to categorize CPSS and not all studies used the same method, we could only classify their cases by their descriptions. Moreover, the patients diagnosed with agenesis of the ductus venosus (ADV) were also included in the DVSS group because they had similar clinical characteristics and CPSS could occur in these patients [31, 33, 43, 45]. Nonetheless, we still believe our study is useful for perinatal management and antenatal counselling of CPSS.

## Conclusion

CPSS is a congenital malformation and has a wide spectrum of comorbidities so the ultrasonic characteristics and outcomes vary greatly. As early diagnosis and close monitoring may influence the outcomes, we propose some suggestions about perinatal management that fetal echocardiography should be conducted when fetuses are diagnosed with CPSS and chromosome examinations or genetic examinations should be performed after the diagnosis is made. Close monitoring is essential for fetuses with USS and neonates with EHPSS. Regarding therapy, personalized treatment should be used. Patients who are asymptomatic and have no severe complications should receive conservative treatment as they may experience spontaneous closure, especially IHPSS patients. IHPSS patients are more likely to have good outcomes so they may benefit from the “wait-and-see” approach. Interventional and surgical treatments are still needed to close shunts when patients have severe complications due to CPSS or when the shunt flow is high.

## Data Availability

The datasets used and/or analyzed during the current study are not openly available due to reasons of privacy and are available from the corresponding author on reasonable request.

## Abbreviations

CPSS: Congenital portosystemic shunt
IUGR: Intrauterine growth restriction
EHPSS: Extrahepatic porto–systemic shunt
IHPSS: Intrahepatic porto–systemic shunt
USS: Umbilical–systemic shunt
DVSS: Ductus venosus–systemic shunt
TOP: Termination of pregnancy
IHPVS: Intrahepatic portal venous system
UPSVS: Umbilical–portal–systemic venous shunt
UV: Umbilical vein
PV: Portal vein
DV: Ductus venosus
